# Resistance to BRAF inhibitors induces glutamine dependency in melanoma cells

**DOI:** 10.1016/j.molonc.2015.08.003

**Published:** 2015-08-20

**Authors:** Franziska Baenke, Barbara Chaneton, Matthew Smith, Niels Van Den Broek, Kate Hogan, Haoran Tang, Amaya Viros, Matthew Martin, Laura Galbraith, Maria R. Girotti, Nathalie Dhomen, Eyal Gottlieb, Richard Marais

**Affiliations:** ^1^ Molecular Oncology Laboratory, Cancer Research UK Manchester Institute, The University of Manchester, Wilmslow Road, Manchester M20 4BX, UK; ^2^ Cancer Metabolism Research Unit, Cancer Research UK Beatson Institute, Switchback Road, Glasgow G61 1BD, UK

**Keywords:** BRAF, Resistance, Melanoma, Metabolism, Glutaminolysis

## Abstract

BRAF inhibitors can extend progression‐free and overall survival in melanoma patients whose tumors harbor mutations in BRAF. However, the majority of patients eventually develop resistance to these drugs. Here we show that BRAF mutant melanoma cells that have developed acquired resistance to BRAF inhibitors display increased oxidative metabolism and increased dependency on mitochondria for survival. Intriguingly, the increased oxidative metabolism is associated with a switch from glucose to glutamine metabolism and an increased dependence on glutamine over glucose for proliferation. We show that the resistant cells are more sensitive to mitochondrial poisons and to inhibitors of glutaminolysis, suggesting that targeting specific metabolic pathways may offer exciting therapeutic opportunities to treat resistant tumors, or to delay emergence of resistance in the first‐line setting.

## Highlights

- We examine the metabolic requirements in melanoma cell lines resistant to BRAF inhibitor PLX4720.
- Cells that are resistant to BRAF inhibitors are more dependent on glutamine than glucose as their carbon source.
- Combined glutaminolysis and BRAF inhibition was effective to promote a delay in the onset of resistance.

## 1 Introduction

One of the hallmarks of cancer is metabolic reprogramming, which provides the nutrients and redox potential needed to support continuous proliferation and growth in environments that are deprived of oxygen and nutrients (Cantor and Sabatini, 2012; Ward and Thompson, 2012). Aerobic glycolysis (the Warburg effect) converts glucose to lactate regardless of oxygen availability and is one of the most common characteristics of solid tumors. In many cases, these traits are driven directly by oncogenes and tumor suppressors, altering glucose and glutamine metabolism in tumor cells (Cantor and Sabatini, 2012). For example, oncogenic BRAF and oncogenic RAS regulate expression of genes in the glycolysis, pentose phosphate and glutaminolysis pathways, changing the dependence of cells on different carbon sources (Haq et al., 2013; Ying et al., 2012).

Melanoma is a heterogenetic disease with multiple subtypes driven by specific genetic alterations. About 45% of cutaneous melanomas harbor mutations in BRAF, a protein kinase that is part of the RAS/RAF/MEK/ERK pathway and which regulates cell proliferation and survival (Solit et al., 2006; Wan et al., 2004). The most common mutation in BRAF is a glutamine for valine substitution at position 600 (V600E), which produces an active kinase that drives constitutive MEK/ERK signaling and cell proliferation (Davies et al., 2002; Dhomen and Marais, 2009; Wan et al., 2004). Drugs that inhibit ^V600E^BRAF, such as vemurafenib and dabrafenib, or drugs that inhibit MEK such as trametinib and cobimetinib can extend overall survival in melanoma patients whose tumors harbor a ^V600E^BRAF mutation (Hauschild et al., 2012; Lito et al., 2013; Menzies and Long, 2013; Sosman et al., 2012). Unfortunately however, intrinsic and secondary/acquired resistance limits the overall response and therapeutic benefit of these personalized medicines (Girotti et al., 2014 Sep, 2010, 2010, 2013, 2013 Mar, 2008, 2010, 2011, 2011, 2012). Many studies have shown that resistance to BRAF targeted therapies is mediated by alterations in the signaling pathways that control cell growth. Up‐regulation of receptor tyrosine kinase signaling, or mutations in NRAS or MEK have all been shown to regulate resistance, as have upregulation or alternative splicing of ^V600E^BRAF itself (Lito et al., 2013). Similarly, increased release of growth factors by the stromal cells, or alterations to transcription factors that control expression of signaling components can mediate resistance (Straussman et al., 2012; Wilson et al., 2012).

In other diseases, it has emerged that metabolic rewiring can also contribute to resistance. For example, lapatinib/trastuzumab‐resistant breast cancer cells display a dependency on glucose metabolism and the ER‐stress network (Komurov et al., 2012; Zhao et al., 2011). Moreover, melanoma cells display aerobic glycolysis and use glutamine to replenish tricarboxylic acid (TCA) cycle metabolites (Hall et al., 2013; Scott et al., 2011). ^V600E^BRAF regulates glycolysis genes and oxidative metabolism in some melanoma cells through the transcription factors MITF and PGC1α, and ERK signaling suppresses the oxidative phenotype (Haq et al., 2013; Vazquez et al., 2013). Furthermore, vemurafenib‐resistant cell lines display increased mitochondrial respiration, rendering them more vulnerable to oxidative stress‐mediated cell death (Corazao‐Rozas et al., 2013). Here we investigate how metabolism is changed in melanomas that develop acquired resistance to BRAF inhibitors to identify new therapeutic targets for patients who relapse on first‐line targeted therapies.

## 2 Material and methods

### 2.1 Cell lines and reagents

General reagents were purchased from Sigma (St. Louis, MO, USA), except PLX4720, which was from 3way Pharm (Shanghai, China). A375, Colo829, SKMEL5 and G361 cells were purchased from the ATCC and maintained in RPMI or DMEM, supplemented with 10% FBS, 1 mM sodium pyruvate, 2 mM glutamine and 1% penicillin/streptomycin. To generate BRAF inhibitor resistant clones cells were cultured in increasing concentrations of PLX4720 (0.1–1 μM) and the resistant clones were maintained in 1 μM PLX4720 thereafter.

### 2.2 Metabolic assays

Oxygen consumption rate was measured with the optical fluorescent oxygen/hydrogen sensor XF^e^96 Seahorse analyzer. Briefly cells (25,000/well) were incubated overnight and washed into unbuffered DMEM with an adjusted pH of 7.4 according to the manufacturer's instructions. The mitochondria stress kit was used to measure OCR responses using the following concentrations: 1 μM oligomycin, 2 μM FCCP, 1 μM rotenone and 1 μM antimycin A. The data was normalized to protein content by sulforhodamine B (SRB) staining.

Lactate secretion was analyzed using Biovison Lactate kits. Briefly cells (25,000/well) were cultured overnight and incubated in glucose‐ and glutamine‐free DMEM/10% FBS plus 1 mM pyruvate for one hour prior to incubation in full medium for one hour. 10 μL supernatant were used for the analysis and raw values were normalized to protein content.

### 2.3 Mitochondrial morphology

Cells (2 × 10^5^ in 6 cm glass‐bottomed plates) were stained with Mitotracker Green (100 nM; Invitrogen) and the nuclei were counter‐stained with Hoechst (5 μg/mL; Invitrogen) and then visualized on a Nikon A1R confocal microscope.

### 2.4 Metabolic fluxes and exchange rates

Cells were incubated for 48 h in full RPMI media and exchange rates for glucose, lactate, pyruvate, glutamine and glutamate were calculated by comparing the peak area for each metabolite in full RPMI media kept under the same conditions for 48 h without cells and considering the average cell number during the culture time. Metabolites in 20 μL culture media were extracted (6 biological replicates per experiment; 3 independent experiments) in 980 μL ice cold acetonitrile:methanol:water (3:5:2), and centrifuged (10 min, 16,000 g) at 4 °C for LC‐MS analysis (Chaneton et al., 2012).

For intracellular labeling experiments, cells were incubated in full RPMI media for 24 h in the presence of 10 mM U‐^13^C_6_ glucose or 2 mM U‐^13^C_5_ glutamine. The cells were washed twice with PBS and intracellular metabolites extracted in acetonitrile:methanol:water (3:5:2) at 1 ml per 10^6^ cells. Samples were analyzed by LC‐MS using a Sequant ZIC‐pHILIC column (2.1 mm × 150 mm, 5 μm polymeric beads, guard column Sequant ZIC‐pHILIC guard peek 2.1 mm × 20 mm, Millipore) using formic acid, water, and acetonitrile as components of the mobile phase. Mass spectrometry was performed in a Thermo Scientific Exactive Benchtop LC/MS Orbitrap Mass spectrometer (Chaneton et al., 2012).

### 2.5 Histology and immunohistochemistry (IHC)

Tumors were formalin‐fixed and analyzed as previously described (Girotti et al., 2013). Sample preparation for mitochondrial staining was performed with antigen retrieval (sodium citrate pH6.0). Positive and negative controls were included in each experiment. Whole faced sections of paired melanoma samples from patients corresponding to tumor tissue before and after vemurafenib treatment were analyzed. Samples were scored blind and a staining score was provided for each section, where score = Σ(% of cells with intensity 4 * 4) + (% of cells with intensity 3 * 3) + (% of cells with intensity 2 * 2) + (% of cells with intensity 1 * 1); where 0 = no staining, 1 = light, 2 = moderate 3 = intense and 4 = very intense staining. The p value (2‐sided) for the Wilcoxon matched‐pairs signed rank test is 0.0313.

### 2.6 siRNA experiments

Melanoma cells were trypsinized and counted (1 × 10^6^ cells/well) and transfected with Lipofectamine 3000 and a non targeting control siRNA (AllStars negative Control #SI03650318, QIAGEN) or two different siRNAs targeting *PGC1α* (Hs_PPARGC1A_2 and 6 FlexiTube siRNA; #SI00101031 and #SI02639833; all from QIAGEN). Two rounds of transfection were performed within 48 h prior to cells being used for RNA analysis or drug response experiments.

### 2.7 Short‐term growth inhibition assays

Cells (10^3^/well) were seeded into 96‐well plates and incubated with various dilutions of PLX4720, BPTES, phenformin, buformin, metformin or 2DG for 72 h. Cell viability was determined by SRB staining and results were normalized to untreated controls after background subtraction.

### 2.8 Long‐term cell proliferation assays

Cells (5 × 10^4^/well) were seeded into 6‐well plates and cultured for 10 days with the indicated drugs and then stained with crystal violet. Quantification was performed by dissolving the crystal violet in 500 μL methanol.

### 2.9 Mouse xenografts

All procedures involving animals were approved by CRUK Manchester Institute's Animal Welfare and Ethical Review Body, in accordance with the Animals (Scientific Procedures) Act 1986, carried out under license PPL/70/7701 and reported according to the NC3Rs ARRIVE guidelines. Five to six week old female nude mice were injected subcutaneously with 1 × 10^6^ A375 or A375/R cells. Tumors were allowed to establish to 100–150 mm^3^, size matched, and then the mice were randomly allocated to groups of 8 animals. No blinding was used in the treatment schedules for these studies. Based on literature precedents, groups of 8 animals were used, to provide sufficient animals per cohort to provide statistically significant data, whilst keeping animal numbers to a minimum. Treatment was administered by oral gavage daily with vehicle (5% DMSO, 95% water) or 45 mpk (mg per kilo) PLX4720. For the glutaminolysis inhibition study, 12.5 mpk BPTES or vehicle was injected intraperitoneally every two days. Tumor size was determined by caliper measurements of tumor length, width, and depth, and volume was calculated as volume = 0.5236 × length × width × depth (mm). In accordance with our license to perform animal experiments, animals were excluded from the experiments if they displayed signs of distress, excessive bodyweight loss (>20%) or illness.

### 2.10 Statistical analysis

Statistics were performed with GraphPad Prism^®^ version 6.0b (GraphPad Software, San Diego, CA, USA). Data are presented as mean ± SD or mean ± SEM. The student's t‐test or Wilcoxon matched‐pairs signed rank test was performed and statistical significance values are ≤0.05.

## 3 Results

### 3.1 BRAF inhibitor resistance is associated with increased mitochondrial biogenesis and oxidative metabolism

**Figure 1:**
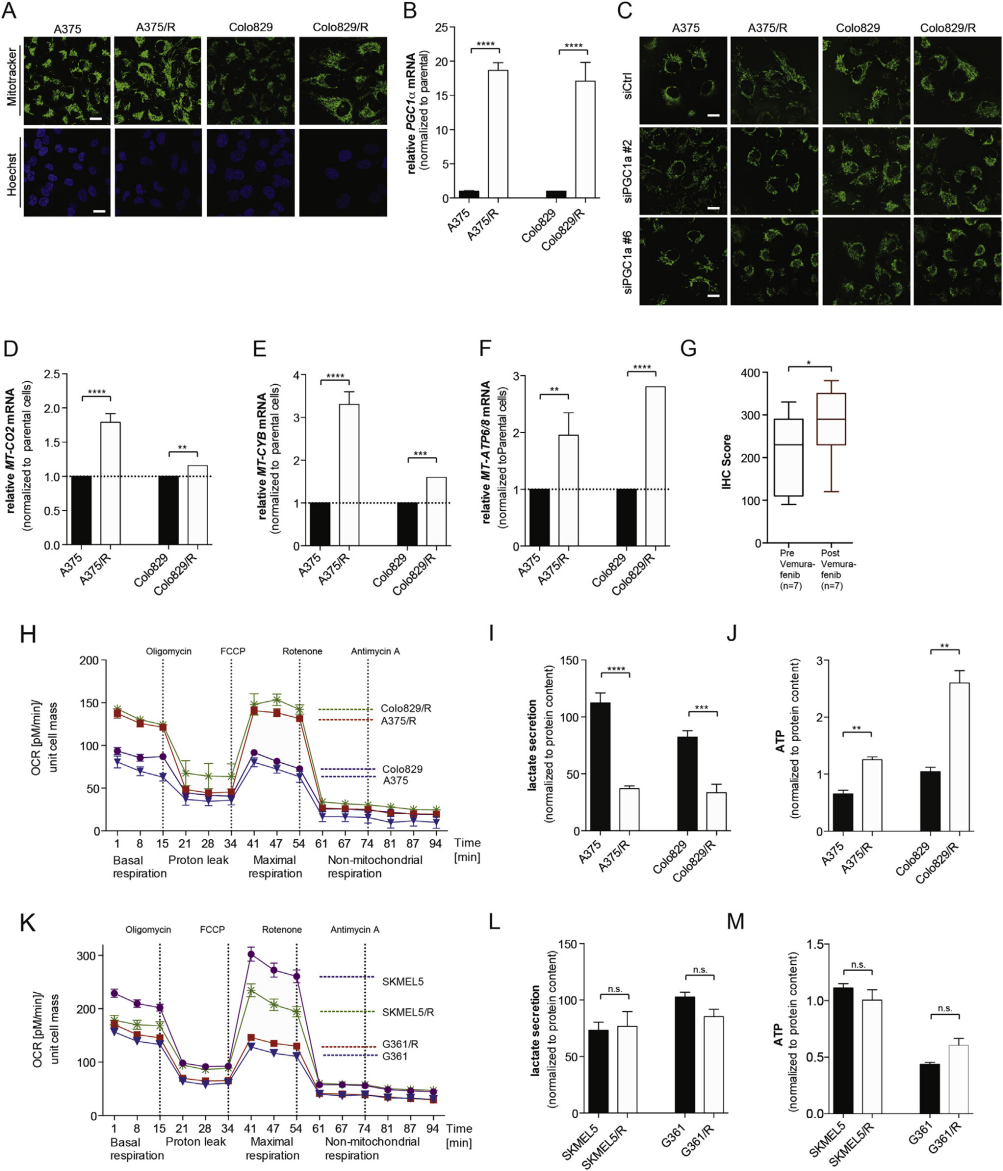
BRAF inhibitor resistant melanoma cells exhibit increased mitochondrial mass and oxidative metabolism. (A) Photomicrographs showing mitochondrial morphology (Mitotracker Green) and nuclei (Hoechst, blue) in A375, Colo829, A375/R and Colo829/R cells. (B)PGC1α levels in A375, Colo829, A375/R and Colo829/R cells. Error bars represent SEM of at least two independent experiments with 3 biological replicates. ****p < 0.0001. (C) Photomicrographs showing mitochondrial morphology (Mitotracker Green) in A375, Colo829, A375/R and Colo829/R cells after silencing with siRNA control (siCtrl) or two different siRNA probes against PGC1α. (D–F)MT‐CO2(D), MT‐CYB(E), and MT‐ATP6/8(F) mRNA levels in A375, Colo829, A375/R and Colo829/R cells. Error bars represent SEM of at least two independent experiments with 3 biological replicates. *p < 0.05, **p < 0.01, ***p < 0.001 and ****p < 0.0001. (G) The box‐plot showing quantification of MT‐CO2 staining in 7 paired samples from patients before and after emergence of vemurafenib resistance; *p < 0.05, and test (Wilcoxon Signed‐Rank Test). (H) Oxygen consumption rate (OCR) changes in response to mitochondrial function modulators in A375, Colo829, A375/R and Colo829/R cells (n = 3). (I–J) Lactate secretion (I) and intracellular ATP levels (J) in A375, Colo829, A375/R and Colo829/R cells. Error bars represent SEM of at least two independent experiments with 3 biological replicates. **p < 0.01, ***p < 0.001, ****p < 0.0001. (K) Oxygen consumption rate (OCR) changes in response to mitochondrial function modulators in SKMEL5, SKMEL5/R, G361 and G361/R cells (n = 3). (L–M) Lactate secretion (L) and intracellular ATP levels (M) in SKMEL5, G361, SKMEL5/R and G361/R cells. Error bars represent SEM of at least two independent experiments with 3 biological replicates. n.s.: not significant.

We have reported that BRAF mutant melanoma cells develop drug resistance when grown in the presence of BRAF inhibitors (Girotti et al., 2013). For the studies reported here, we used BRAF mutant A375 and Colo829 melanoma cell clones (A375/R and Colo829/R respectively) that were over 100‐fold less sensitive to the BRAF inhibitor PLX4720 than their respective parental cells (Supplemental Figure 1A). Staining with MitoTracker Green revealed that the mitochondria in the resistant cells were elongated compared to those in the parental cells (Figure 1A) and we found that the resistant cells were more sensitive than the parental cells to the biguanide mitochondrial poisons phenformin, metformin and buformin (Supplemental Figure 1B). Thus BRAF inhibitor resistant cells displayed altered mitochondrial morphology and increased dependence on mitochondrial function, so we examined metabolism in these cells.

The resistant cells displayed increased expression of *PGC1α* (Figure 1B), a transcription coactivator that regulates mitochondrial biogenesis (Puigserver and Spiegelman, 2003). We show that depletion of *PGC1α* by siRNA (Supplemental Figure 1C) reversed mitochondrial elongation in the resistant cells, but did not affect mitochondrial morphology in the parental cells, linking *PGC1α* expression to the altered mitochondrial morphology (Figure 1C, Supplemental Figure 1D). Consistent with increased mitochondrial biogenesis, we show increased expression of the mitochondrial respiratory chain genes *MT‐CO2*, *MT*‐*CYB*, and *MT‐ATP6/8* in the resistant cells (Figure 1D–F) and confirmed that MT‐CO2 protein expression was increased in the resistant cells (Supplemental Figure 2A, B). Critically, we show that MT‐CO2 expression was increased in melanomas from 7 patients who presented resistance to vemurafenib (p = 0.03; Figure 1G, Supplemental Figure 2C), demonstrating the clinical relevance of our findings.

Mitochondrial elongation is associated with increased oxidative metabolism (Gomes et al., 2011) and we show that basal and maximal respiration of A375/R and Colo829/R cells were substantially increased compared to their drug‐sensitive parental cells (Figure 1H). We also observed decreased lactate secretion (Figure 1I) and increased intracellular ATP (Figure 1J). Note that the increased intracellular ATP was not due to increased proliferation, as the resistant cells actually grew more slowly than the parental cells (Supplemental Figure 2D).

Next, we examined these responses in SKMEL5 and G361 cells, because these BRAF mutant melanoma cells are intrinsically resistant to BRAF inhibitors and are ∼10 fold less sensitive to PLX4720 than A375 and Colo829 (Supplemental Figure 2E). Accordingly, continual exposure of SKMEL5 and G361 cells to PLX4720 only modestly affected their sensitivity to this compound (Supplemental Figure 2E), did not cause increased basal or maximal respiration (Figure 1K) and did not cause decreased lactate secretion or increased intracellular ATP (Figure 1L, M). Thus, PLX4720 did not alter oxidative metabolism in intrinsically resistant cells.

### 3.2 BRAF inhibition reduces glycolytic flux

**Figure 2:**
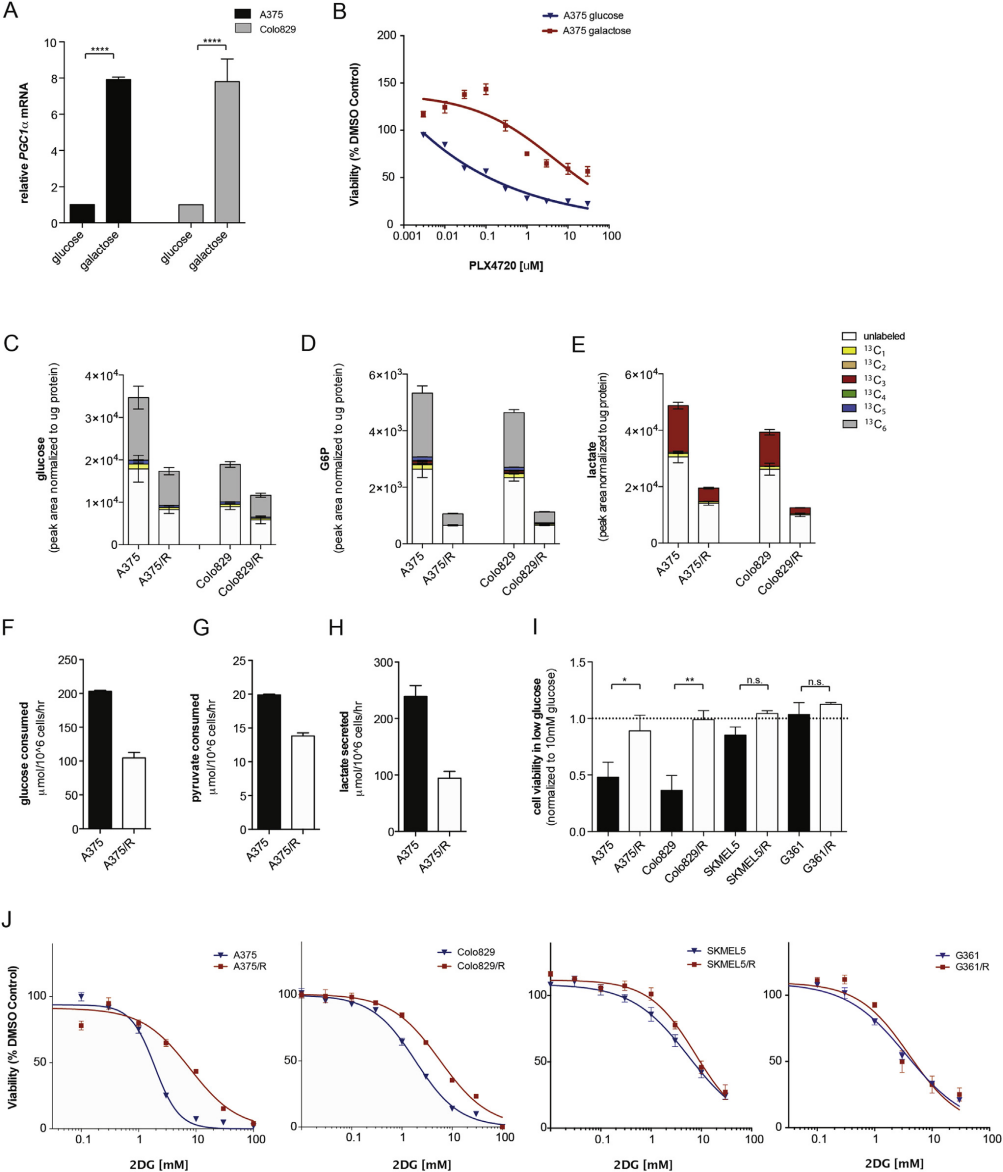
BRAF inhibitor resistant melanoma cells display decreased glycolytic flux. (A) Graph showing PGC1α mRNA levels in A375 and Colo829 cells grown for 5 days in medium containing glucose or galactose as the sole carbon source. Error bars represent SEM of at least two independent experiments with 3 biological replicates. ****p < 0.0001. (B) Graph showing A375 cell growth (sulforhodamine B) in glucose or galactose containing medium in the presence of increasing concentrations of PLX4720 for 72 h. (C–E) Graphs showing glucose (C), glucose‐6‐phosphate (D), and lactate (E) levels in A375, Colo829, A375/R and Colo829/R cells labeled with U‐13C6 glucose. The graphs show mean ± SD (n = 6) and similar results were observed in two independent experiments. Legend displays the different isotopomers. 13C1: one carbon atom labeled; 13C2 – 13C6: two to six carbon atoms labeled. Glucose and glucose‐6‐phosphate are 6‐carbon molecules hence 13C6 labeling (gray). Lactate is a 3‐carbon molecule hence the 13C3 labeling (red). Endogenous metabolites are unlabeled and shown in white. (F–H) Graphs showing glucose (F), pyruvate, (G) and lactate (H) consumption (extracellular exchange rate) in A375 and A375/R cells. Data are representative of three independent studies and displayed as mean ± SD of n = 6. (I) Growth (sulforhodamine B) of A375, A375/R, Colo829, Colo829/R, SKMEL5, SKMEL5/R, G361 and G361/R cells in medium containing 1 mM glucose for 72 h. Data are expressed relative to growth in medium containing 10 mM glucose (dotted line). Error bars represent SEM of at three independent experiments with 3 biological replicates. *p < 0.05, **p < 0.01, n.s.: non‐significant. (J) Growth of A375, A375/R, Colo829, Colo829/R, SKMEL5, SKMEL5/R, G361 and G361/R cells in the presence of increasing concentrations 2DG for 72 h (n = 3).

The data above show that BRAF inhibitor‐resistant cells present increased mitochondrial mass, increased dependence on mitochondrial function and increased oxidative respiration. Consistent with these observations, when parental A375 and Colo829 cells were forced to depend on oxidative metabolism by culturing in galactose as the only carbon source, *PGC1α* expression increased (Figure 2A) and the cells were more resistant to PLX4720 (Figure 2B). Thus, switching to oxidative metabolism mediates resistance to BRAF inhibitors, so we examined glucose consumption in the resistant cells. We added uniformly labeled ^13^C (U‐^13^C) glucose to the cells and measured intracellular metabolites and observed lower levels of glucose in the resistant than parental cells (Figure 2C). Similarly, we detected lower levels of intracellular glucose‐6‐phosphate (Figure 2D) and lactate (Figure 2E) in the resistant cells and accordingly, the resistant cells consumed less glucose and pyruvate, and they secreted less lactate (Figure 2F–H). Critically, the A375/R and Colo829/R cells were more tolerant of glucose starvation and less sensitive to inhibition of glycolysis by the small molecule inhibitor 2‐deoxyglucose (2DG; Figure 2I, J). Note that the SKMEL5/R and G361/R cells were no less sensitive to glucose starvation and no more resistant to 2DG (Figure 2I, J) than their parental cells, demonstrating that these effects do not occur in intrinsically resistant cells.

### 3.3 PLX4720‐resistant cells display increased glutaminolysis

**Figure 3:**
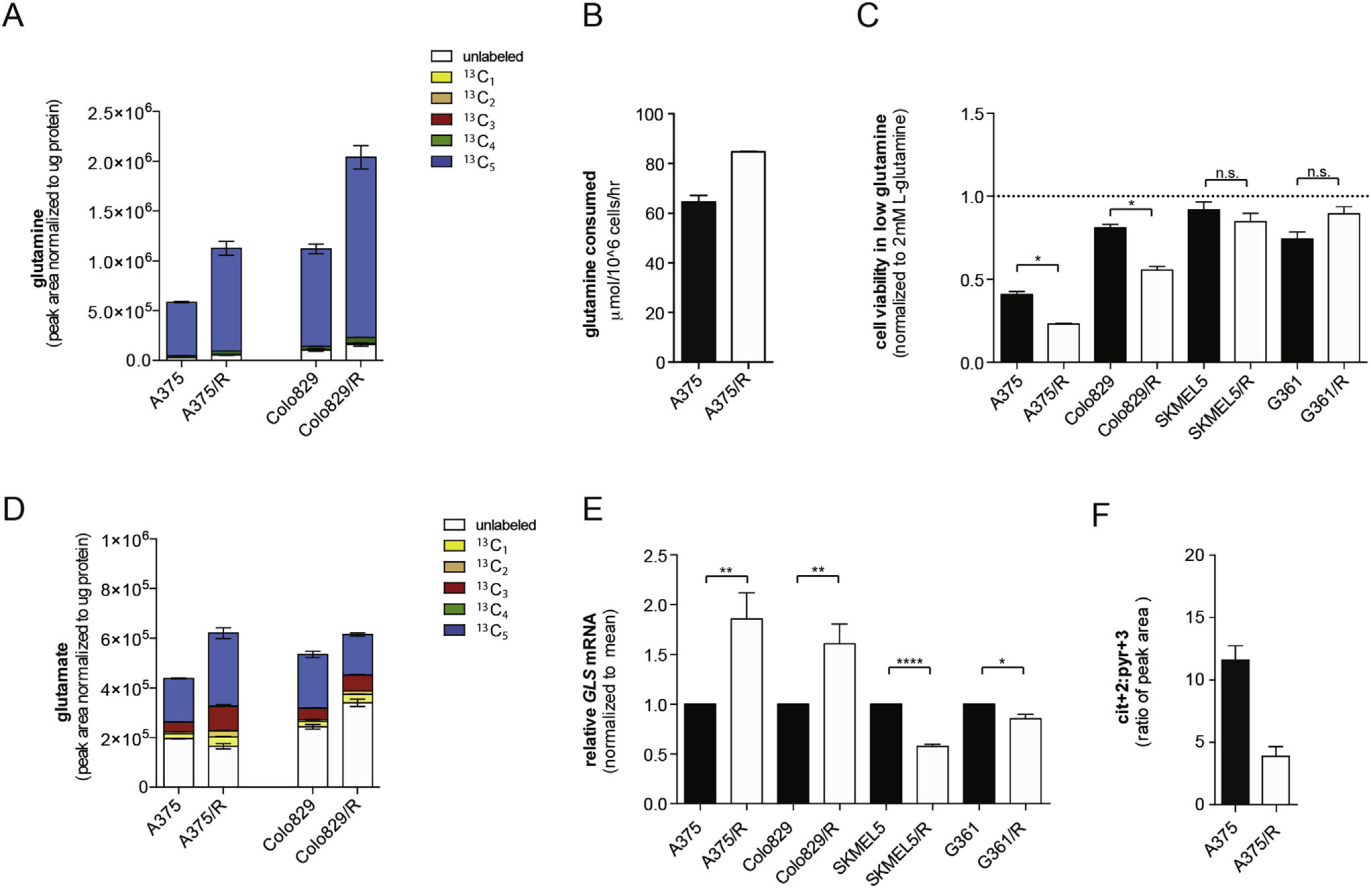
BRAF inhibitor resistant melanoma cells show increased glutamine metabolism. (A) Graph showing intracellular glutamine levels in A375, A375/R, Colo829 and Colo829/R cells labeled with U‐13C5 glutamine. Legend displays isotopomer distribution. 13C1: one carbon atom labeled; 13C2–13C5: two to five carbon atoms labeled. Glutamine is a 5‐carbon molecule hence 13C5 labeling (blue). Endogenous glutamine is unlabeled and shown in white. (B) Graph showing glutamine consumption (extracellular exchange rates) in A375 and A375/R cells. Data are representative of three independent studies and displayed as mean ± SD of n = 6. (C) Graph showing growth (sulforhodamine B) of A375, A375/R, Colo829, Colo829/R, SKMEL5, SKMEL5/R, G361 and G361/R cells in medium containing 0.5 mM glutamine for 72 h. Data are presented relative to medium containing 2 mM glutamine. Error bars represent SEM of three independent experiments with 3 biological replicates. *p < 0.05, ns: non‐significant. (D) Graph showing intracellular glutamine levels in A375, A375/R, Colo829 and Colo829/R cells labeled with U‐13C5 glutamine. Data are a representative experiment of mean ± SD of n = 6. Similar results were observed in two independent experiments. Legend displays isotopomer distribution. 13C1: one carbon atom labeled; 13C2 to 13C5: two to five carbon atoms labeled. Glutamate is a 5‐carbon molecule hence 13C5 labeling (blue). Endogenous glutamate is unlabeled and shown in white. (E) Graph showing GLS mRNA levels in A375, A375/R, Colo829, Colo829/R, SKMEL5, SKMEL5/R, G361 and G361/R cells. Three independent experiments with n = 3 were performed and results display mean ± SEM. *p < 0.05, **p < 0.01, and ****p <0.0001. (F) Ratio of intracellular citrate (Cit+2) to pyruvate (Pyr+3) in A375 and A375/R cells.

Thus, resistant cells are less dependent on glucose for proliferation, and since recent studies have established that glutamine is an important alternative carbon source for tumor growth, we examined glutamine dependency in these cells. To analyze the glutamine carbon flux, we incubated the cells with uniformly labeled ^13^C (U‐^13^C) glutamine and show that intracellular glutamine was increased in the resistant cells (Figure 3A). Consistently, the resistant cells consumed more glutamine (Figure 3B) and were more sensitive to glutamine starvation (Figure 3C). Note however that the SKMEL5/R and G361/R cells were no more sensitive to glutamine starvation than their parental clones (Figure 3C).

These data suggest that cells with acquired resistance are more reliant on glutamine, and accordingly we observed increased glutamate levels in resistant cells (Figure 3D). Consistent with this, we observed increased expression of *GLS*, a glutaminase that converts glutamine to glutamate (Supplemental Figure 3A), in the resistant cells (Figure 3E). Note that GLS expression did not increase, but actually decreased in SKMEL5/R and G361/R cells (Figure 3E). Commensurate with these results, we detected a lower contribution from glucose to citrate (identified by the changes in the levels of M+3 mass isotopomer of pyruvate (Pyr+3) and of the M+2 mass isotopomer of citrate (Cit+2) using U‐^13^C glucose) in the resistant cells (Figure 3F).

### 3.4 Inhibition of glutaminolysis blocks the growth of PLX4720‐resistant tumors

**Figure 4:**
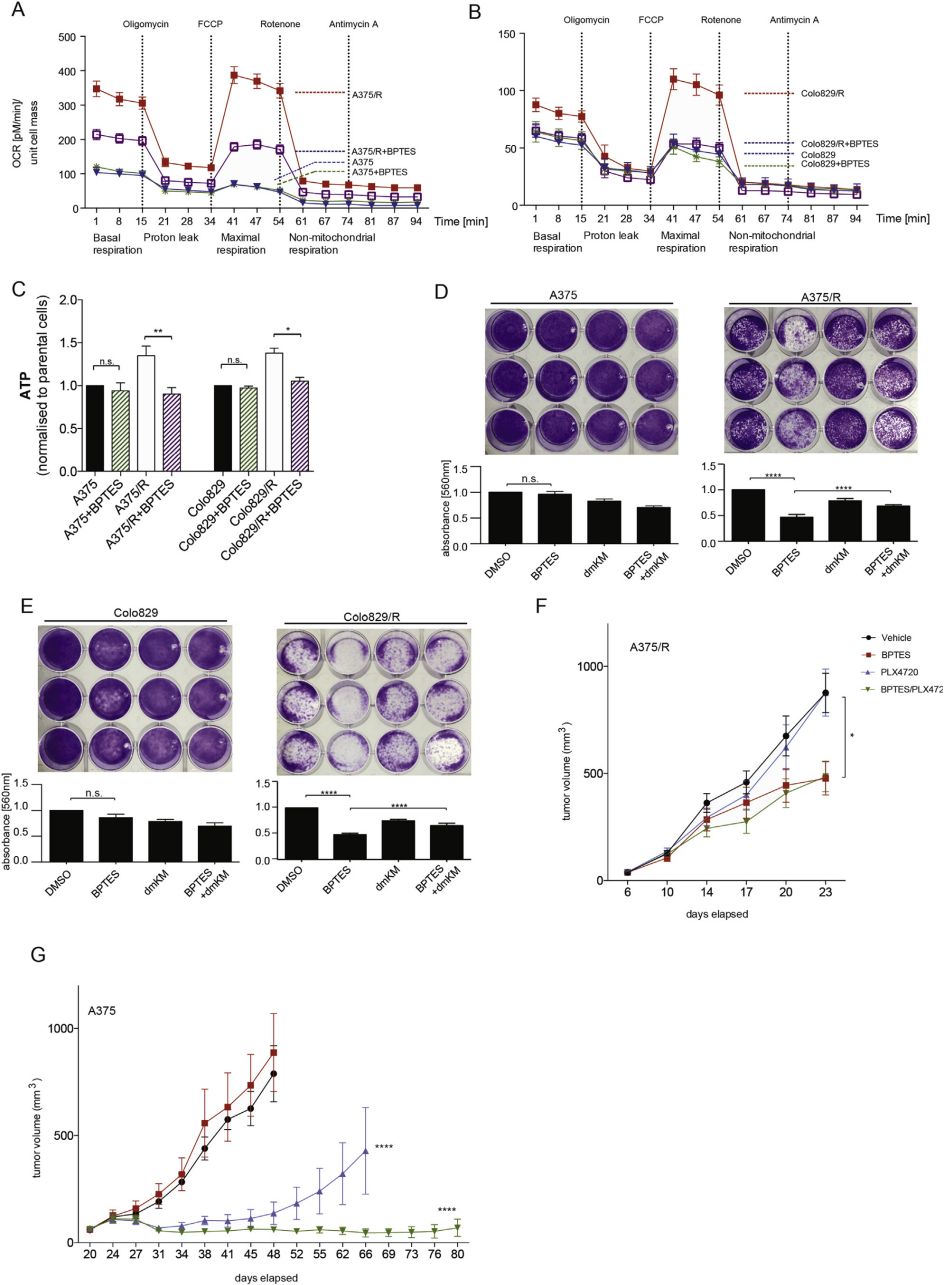
Glutaminolysis inhibition diminishes oxidative metabolism and cell viability of BRAF inhibitor resistant melanoma cells. (A, B) Oxygen consumption rate (OCR) changes in response to mitochondrial function modulators in A375 and A375/R cells (A), and in Colo829 and Colo829/R cells (B), treated with DMSO (control) or BPTES (2 μM) for 18 h. Raw values were normalized to protein content (n = 3). (C) Graph showing ATP levels in A375, A375/R, Colo829 and Colo829/R cells treated with DMSO (control) or BPTES (2 μM). Error bars represent SEM of at least two independent experiments with three biological replicates each. *p < 0.05, **p < 0.01, n.s.: not significant. (D, E) Long term (10 days) growth of A375 and A375/R cells (D) and in Colo829 and Colo829/R (E) cells in the presence of DMSO (control), BPTES (2 μM), dimethyl‐α‐KG (dmKM; 5 mM) or both. The graphs below the images show quantification by crystal violet. Error bars represent SEM of at least two independent experiments with three biological replicates each; ****p <0.0001 and n.s.: not significant. (F) Graph showing growth of A375/R xenografts in nude mice (n = 8 per group) treated with PLX4720 (45 mpk, p.o. daily) or BPTES (12.5 mpk, IP every second day) or both. Mann–Whitney‐U test *p < 0.05. (G) Graph showing growth of A375 xenografts in nude mice (n = 8 per group) treated with PLX4720 (45 mpk, p.o. daily) or BPTES (12.5 mpk IP every second day) or both. Mann–Whitney‐U test ****p < 0.0001.

Thus, glutamine is a major carbon source for cells with acquired resistance, so we treated the cells with BPTES (Bis‐2‐(5‐phenylacetamido‐1,2,4‐thiadiazol‐2‐yl)ethyl sulfide), a small molecule GLS inhibitor. BPTES did not affect oxygen consumption rate (OCR) in parental cells but significantly reduced basal and maximal respiration in A375/R and Colo829/R cells (Figure 4A, B). Furthermore, BPTES reduces ATP levels in resistant but not parental cells (Figure 4C). Commensurate with these findings, the *in vitro* growth of the parental cells was not affected by BPTES, whereas BPTES inhibited the growth of the resistant cells (Figure 4D, E). Notably, the growth inhibitory effects of BPTES on the resistant cells were partially rescued by dimethyl‐α‐ketoglutarate, a cell‐permeable α‐ketoglutarate analogue that enters the glutaminolysis pathway below the level of GLS (Figure 4D, E; Supplemental Figure 3A). This confirms that the growth‐inhibitory effects of BPTES were mediated by inhibition of glutaminolysis.

Next, we tested BPTES *in vivo* by growing A375/R cells as xenografts in immunocompromised mice. The A375/R tumors were insensitive to PLX4720, and although their growth was delayed by BPTES, PLX4720 and BPTES did not cooperate to inhibit their growth any further (Figure 4F). We next tested BPTES in parental A375 xenografts. In accordance with our *in vitro* results, A375 tumors were insensitive to BPTES, but their growth was delayed by PLX4720 (Figure 4G). Importantly, BPTES enhanced the anti‐tumor activity of PLX4720 against parental A375 tumors, by not only inducing regression, but by also suppressing the emergence of resistance.

## 4 Discussion

It has previously been reported that ^V600E^BRAF inhibition in melanoma cells suppresses expression of glycolytic enzymes, leading to reduced glucose consumption and growth inhibition (Parmenter et al., 2014). We show that increased oxidative metabolism contributes to resistance to BRAF inhibitors, an observation also reported by Corazao‐Rozas et al. (Corazao‐Rozas et al., 2013), suggesting that these drugs place cancer cells under selective pressure to restore oxidative metabolism and hence proliferation. Ectopic expression of ^Q61K^NRAS in the presence of BRAF inhibitors restores expression of glycolytic enzymes in ^V600E^BRAF melanoma cells (Parmenter et al., 2014), but we show that melanoma cells that acquired resistance through continuous exposure to BRAF inhibitors, are less dependent on glucose, but rather switch to glutamine as a major carbon source. Specifically, we show that glutamine uptake is increased and GLS is upregulated. The shift to glutamine metabolism appears to allow the resistant cells to sustain survival and proliferation despite reduced flux of glucose‐derived carbon into the TCA cycle (Metallo et al., 2012; Vander Heiden et al., 2011). Thus, glutaminolysis effectively sustains TCA cycle metabolite levels (anaplerosis) and presumably provides nitrogen for nucleotide biosynthesis.

An increase in glutamine consumption may also provide other advantages. It was recently reported that highly invasive ovarian cancer cells are more dependent on glutamine for survival than cells that present low invasion (Yang et al., 2014) and we recently found that BRAF inhibitor resistant melanoma cells are more invasive than sensitive cells (Sanchez‐Laorden et al., 2014). Thus, the resistant cells may switch to glutamine not only to sustain proliferation; it may also contribute to other hallmarks of cancer such as invasion and metastasis.

Our findings are consistent with studies showing that cancer cells, including melanoma, display metabolic flexibility and can adapt to new/stressful growth conditions (Cantor and Sabatini, 2012; Dang et al., 2009; Frezza et al., 2011; Scott et al., 2011). They are also consistent with data showing that *PGC1α* expression and oxidative phosphorylation are elevated in some ^V600E^BRAF mutant melanomas (Vazquez et al., 2013) and that BRAF inhibition increases *PGC1α* expression in melanoma cell lines (Haq et al., 2013). PGC1α is a key regulator of mitochondrial biosynthesis and *PGC1a* is constitutively upregulated in resistant cells. This is coincident with mitochondrial elongation, increased mitochondrial mass and increased oxidative phosphorylation, and accordingly, the cells show increased dependence on the mitochondria for survival. Thus, in line with another study demonstrating that PLX4720‐resistant melanoma cell lines are more sensitive to metformin and phenformin (Yuan et al., 2013), our data suggests that directly targeting the mitochondria could offer therapeutic opportunities in resistant tumors.

Critically, the resistant cells are less sensitive to glucose starvation and inhibition of glycolysis, but more sensitive to glutamine starvation and inhibition of glutaminolysis. Inhibition of glutaminolysis can suppress the growth of Burkitt's lymphoma and other cancers driven by MYC (Le et al., 2012; Wise et al., 2008; Xiang et al., 2015), and here we show that BRAF inhibitor resistant melanoma cells are also more sensitive to glutaminase inhibition, suggesting that glutaminase may also be a therapeutic target in the resistant tumors. PGC1α is important for glutamine metabolism in *ERBB2*‐positive breast cancer (McGuirk et al., 2013) and glutamine transporters are proposed therapeutic targets in melanoma (Wang et al., 2014), highlighting the therapeutic potential of this metabolic pathway. Accordingly, we show that resistant cells remain sensitive, albeit weakly, to BPTES, but more importantly that BPTES enhances the anti‐tumor activity of BRAF inhibition, presumably by suppressing switching to glutamine metabolism.

Developing effective treatments to delay or overcome resistance in melanoma is a clinical and biological challenge due to the complexity of the multiple mechanisms of resistance that sustain MAPK/ERK signaling in the resistant cells. Recent studies have shown that BRAF regulates metabolism (Corazao‐Rozas et al., 2013; Hall et al., 2013; Parmenter et al., 2014) and our results here show that a subset of melanoma cells that develop resistance to BRAF inhibitors switch from a glycolytic to oxidative phenotype and use glutamine as a major carbon source. We posit that combining BRAF inhibitors with drugs that target glutaminolysis or mitochondrial function may be an effective strategy to treat or prevent resistance to BRAF inhibitors in melanoma patients.

## Contributions

F.B., B.C., M.M, N.D., E.G. and R.M. conceived the project, analyzed the data and wrote the manuscript. F.B. and B.C. performed all *in vitro* experiments together with L.G. and H.T. who also provided microscopy images and F.B. carried out the *in vivo* experiments. F.B, N.v.B and B.C. performed metabolic experiments. N.v.B. performed LC–MS analyses. B.C, helped with metabolic analyses. A.V. analyzed the tumor tissue staining. M.R.G. collected patient tumor samples. F.B., M.S. and K.H. performed the *in vivo* experiments. All authors discussed the results and commented on the manuscript. F.B. and B.C. contributed equally to this work.

## Conflict of interest disclosure statement

The authors declare no conflict of interest in this work/study.

## Supporting information



The following are the supplementary data related to this article:

Supplementary data

Supplementary data

## Supplementary data 1

Supplementary data related to this article can be found at http://dx.doi.org/10.1016/j.molonc.2015.08.003.
