# Supplementary figures and images for: Resistance to BRAF inhibitors induces glutamine dependency in melanoma cells
Source: Mol Oncol. 2015 Aug 20;10(1):73–84. doi: 10.1016/j.molonc.2015.08.003 (PMC4717845; doi:10.1016/j.molonc.2015.08.003)

# Supplemental Figure 1

A

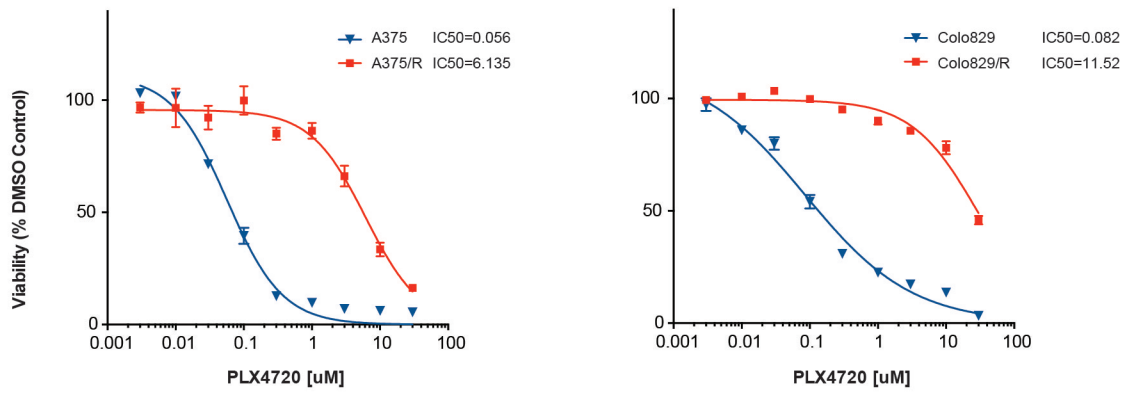

B

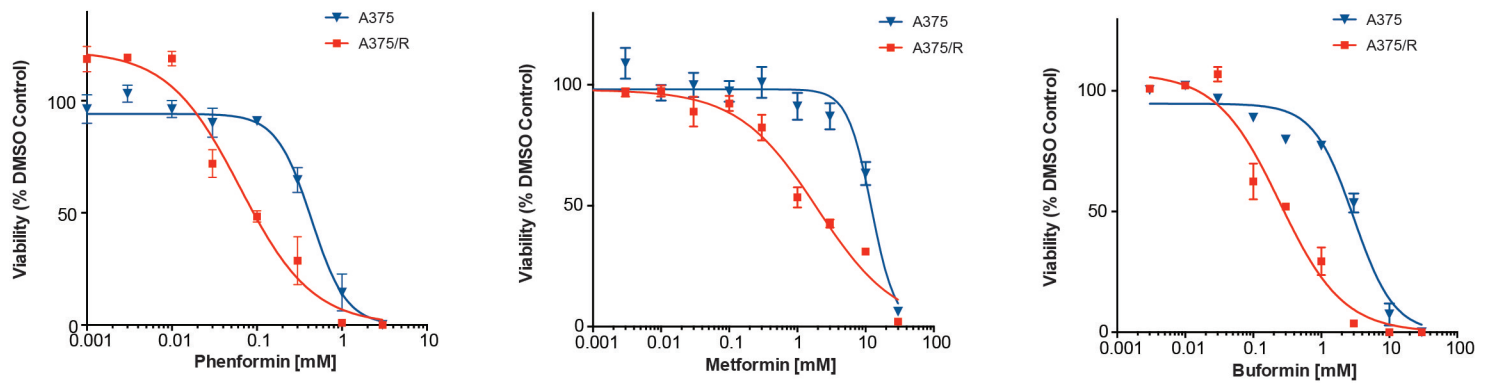

C

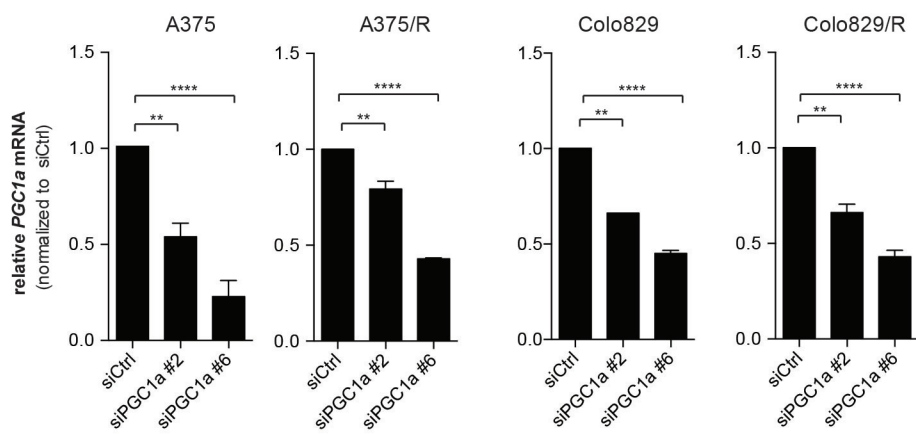

D

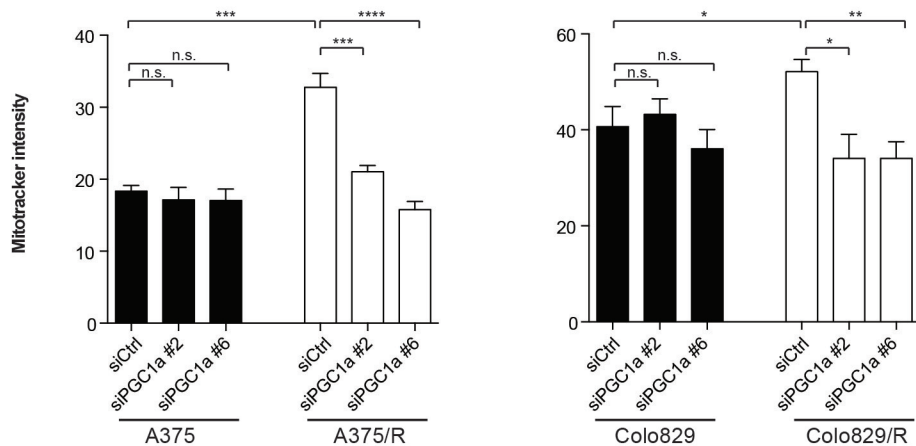

# Supplemental Figure 2

A

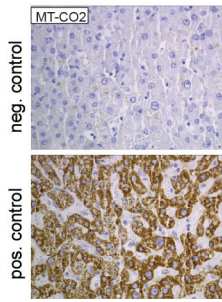

B

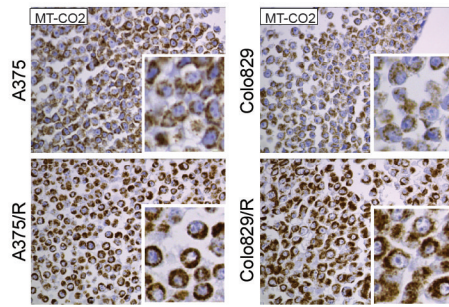

C

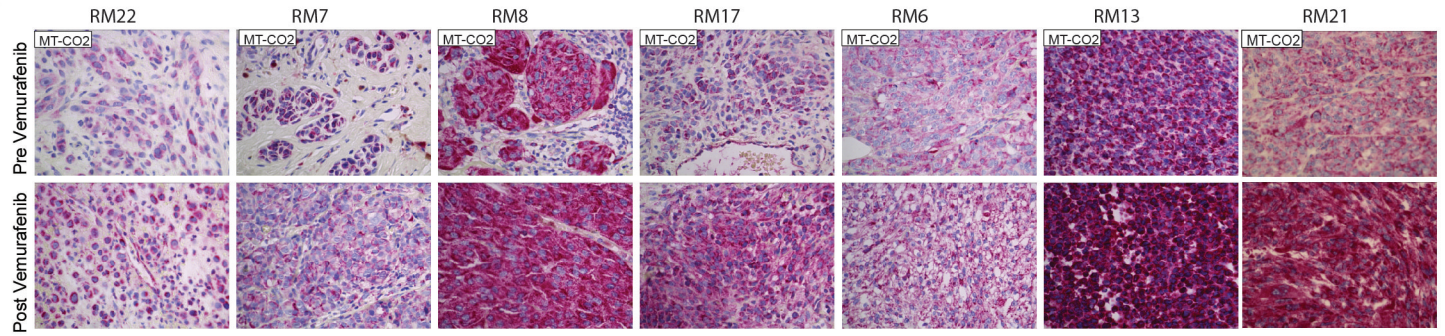

D

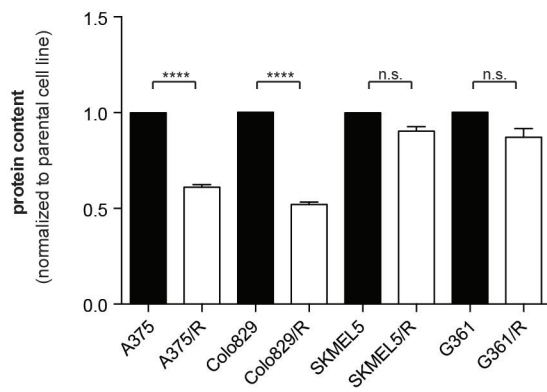

E

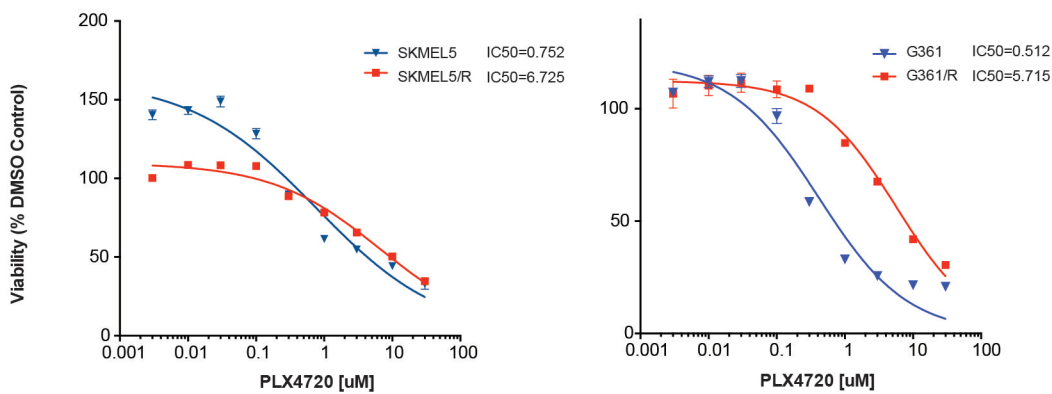

Supplemental Figure 3

A

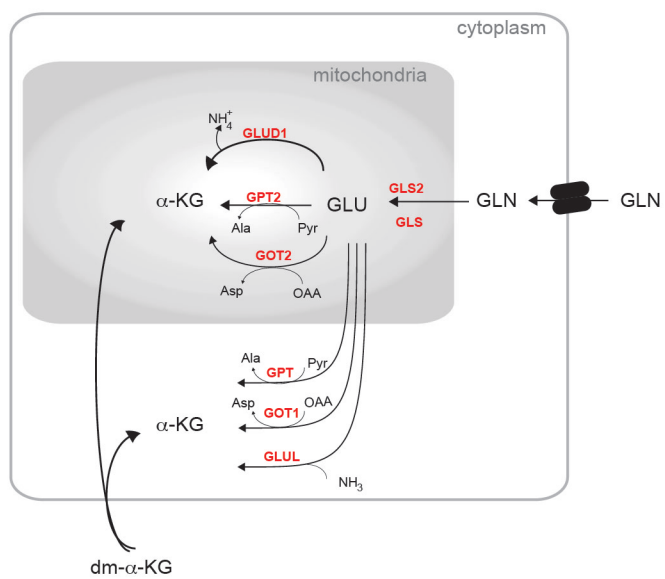

B

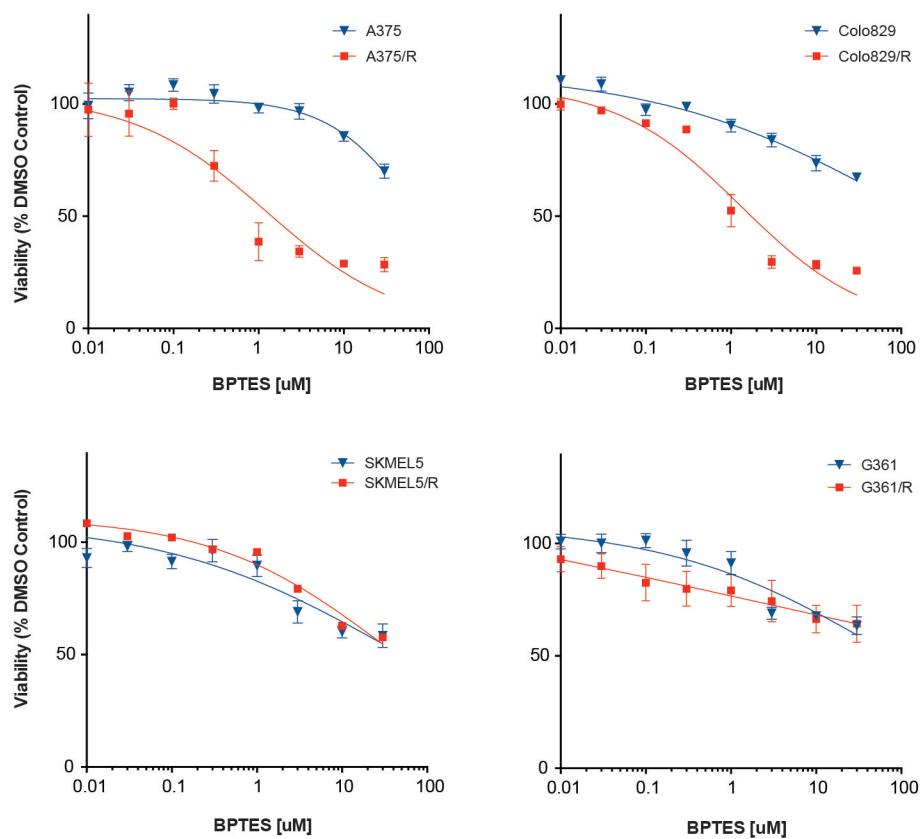

Supplement: Supplementary file 1 — Supplementary data [file MOL2-10-073-s001.pdf]
