# Supplementary material for: Resistance to BRAF inhibitors induces glutamine dependency in melanoma cells
Source: Mol Oncol. 2015 Aug 20;10(1):73–84. doi: 10.1016/j.molonc.2015.08.003 (PMC4717845; doi:10.1016/j.molonc.2015.08.003)
Supplement: Supplementary file 2 — Supplementary data [file MOL2-10-073-s002.pdf]

Supplemental Table 1

Cell line	ATCC number	Disease	Sex	Tissue	Source	Authentication method	Date of authentication	Used after authentication (per frozen vial)
A375	CRL-1619	malignant melanoma	F	skin	ATCC	STR	25.03.2013	< 3 months
A375/R	CRL-1619	malignant melanoma	F	skin	ATCC	STR	25.03.2013	< 3 months
Colo829	CRL-1974	melanoma	M	skin	ATCC	STR	13.03.2013	< 3 months
Colo829/R	CRL-1974	melanoma	M	skin	ATCC	STR	13.03.2013	< 3 months
G361	CRL-1424	malignant melanoma	M	skin	ATCC	STR	06.11.2014	< 3 months
G361/R	CRL-1424	malignant melanoma	M	skin	ATCC	STR	06.11.2014	< 3 months
SKMEL5	HTB-70	malignant melanoma	F	skin	ATCC	STR	06.11.2014	< 3 months
SKMEL5/R	HTB-70	malignant melanoma	F	skin	ATCC	STR	06.11.2014	< 3 months
